# Supplementary material for: A Global Screen for Assembly State Changes of the Mitotic Proteome by SEC-SWATH-MS
Source: Cell Syst. 2020 Feb 26;10(2):133–155.e6. doi: 10.1016/j.cels.2020.01.001 (PMC7042714; doi:10.1016/j.cels.2020.01.001)

Q9P0V3 | SH3B4_HUMAN | SH3BP4 BOG25 EHB10 TTP
Monomer MW [kDa]: 107.496 Monomer expected elution fraction: 40

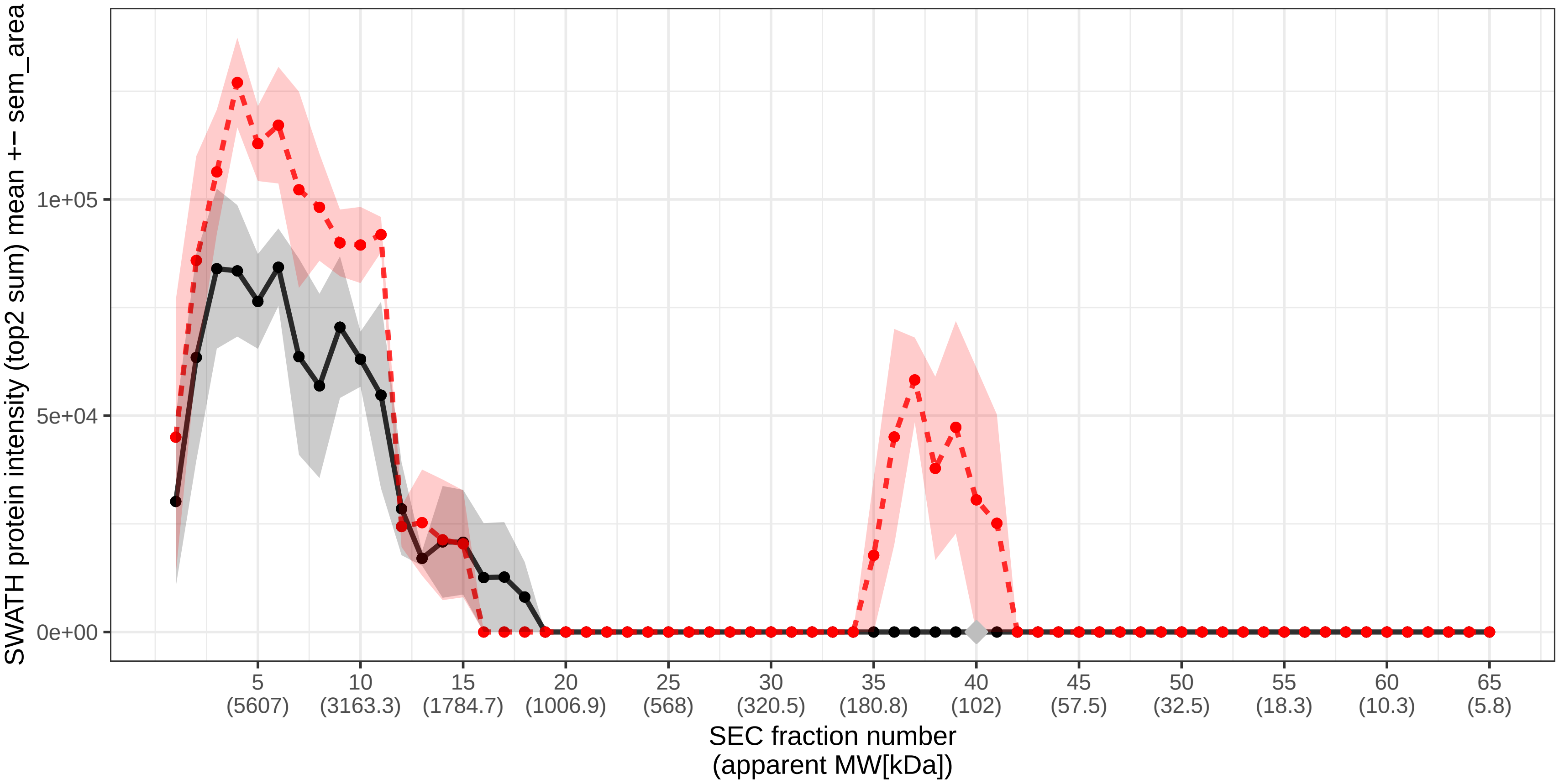

Supplement: Data S1. SEC-SWATH-MS Protein Chromatograms, Related to Figure 1 [file mmc6.zip › SECchrom_Q9P0V3_SH3B4_HUMAN_SH3BP4_BOG25_EHB10_TTP.pdf]
